# A Tailored Motivational Messages Library for a Mobile Health Sleep Behavior Change Support System to Promote Continuous Positive Airway Pressure Use Among Patients With Obstructive Sleep Apnea: Development, Content Validation, and Testing

**DOI:** 10.2196/18793

**Published:** 2020-08-12

**Authors:** Sarah Alismail, Lorne Olfman

**Affiliations:** 1 Claremont Graduate University Claremont, CA United States

**Keywords:** obstructive sleep apnea, mHealth, tailored messages, CPAP therapy, extended parallel process model

## Abstract

**Background:**

Continuous positive airway pressure (CPAP) therapy is the most effective treatment for obstructive sleep apnea (OSA). Despite the reported benefits of CPAP therapy in treating OSA, its effectiveness is reduced by less-than-optimal adherence or use. Up to 50% of patients who accept CPAP therapy fail to adhere to it. As a lack of commitment to CPAP therapy is one of the most significant factors that hinder OSA treatment effectiveness, patient motivation and education are critical to help alleviate the problem of poor CPAP adherence or use.

**Objective:**

This study aims to support the development of mobile health interventions or information systems solutions to promote CPAP adherence and use among patients with OSA through development, content validation, and testing of tailored motivational messages.

**Methods:**

In phase 1, an initial library of 60 messages was developed to promote CPAP use among patients with OSA. In phase 2, draft messages were evaluated for content validation testing for relevance and clarity by research and clinical experts. In phase 3, patients with OSA (N=24) were recruited through a Qualtrics panel to rate the perceived persuasiveness of the messages in terms of threat and efficacy perceptions, as per their computed extended parallel process model (EPPM) response states. The average score of the ratings was calculated for each message. The messages were sorted according to their average (from highest to lowest) to select the best 12 messages for each tailored set based on the potential responses from the EPPM.

**Results:**

In phase 1, 60 messages were developed based on the existing literature and a review of existing materials. In phase 2, the enumerated content validity of the messages was established through the use of the content validity index for items. A total of 57 messages were found to have acceptable content relevance and clarity. In phase 3, patients with OSA perceived the final library of 48 messages to be persuasive.

**Conclusions:**

After the process of content validation and testing, the final library of messages met the criteria for clarity, relevance, and perceived persuasiveness. This study emphasizes the importance of developing and validating the content of motivational messages, grounded in EPPM theory, across the 4 possible response states in terms of high or low efficacy and threat perceptions.

## Introduction

### Background

Obstructive sleep apnea (OSA) is the most common breathing disorder that occurs during sleep in the United States [1], with 30 million adults affected by OSA, which is the second highest occurrence in the world [2,3]. Effective treatment of OSA is essential because untreated OSA has consequences for both individual health and society. These consequences include, but are not limited to, cognitive and behavioral deficits that affect a person’s work performance, including excessive daytime sleepiness, as well as cardiovascular and metabolic dysfunction. Thus, OSA accounts for a significant portion of health care costs, either directly or by means of its associated illnesses and diseases [4]. In the United States alone, the estimated total societal-level cost attributed to OSA is reported to be US $160 billion annually [5].

Continuous positive airway pressure (CPAP) therapy is the most effective treatment for OSA [6,7]. CPAP therapy has been found to improve cognitive processing and daily functioning; reduce daytime sleepiness [8]; and reduce health care costs, physician visits, and work absences [5]. Despite the reported benefits of CPAP therapy in treating OSA, its effectiveness is reduced by less-than-optimal adherence/usage.

Up to 50% of patients who accept CPAP therapy fail to adhere to it [8]. Patients’ failure to adhere to CPAP therapy is a major factor limiting its potential benefits [6,9]. Continued use of CPAP is fundamentally important for effective treatment of OSA [10]. As the lack of commitment to CPAP therapy is one of the most significant factors contributing to hindering the effectiveness of OSA treatment, patient motivation and education about their condition and treatment are critical to help alleviate the problem of poor CPAP adherence. Evidence suggests that behavioral interventions, along with education designed to improve patient self-efficacy, are promising strategies to improve adherence [8]. Thus, finding ways to improve CPAP usage is essential to improving the health and well-being of patients with OSA and might be a way to minimize the associated health care costs and adverse outcomes of the condition [3].

To support future studies on interventions for promoting CPAP use or adherence among patients with OSA, this study aimed to describe the process and results of developing, validating the content of, and testing a set of theory-based tailored motivational messages to be used in a mobile health (mHealth) sleep behavioral change support intervention that promotes CPAP use among patients with OSA.

### Theoretical Framework

The extended parallel process model (EPPM) was developed by Witte [11] as a message design–related theoretical framework for effective health communication aimed at persuading target populations to engage in healthy behaviors [12]. The model considers both emotional and cognitive factors related to message processing and links these processes to the success or failure of a fear appeal [13].

On the basis of the EPPM, individuals are motivated to perform an action through their perception of a threat rather than the actual threat modeled [12]. Perceived threat encompasses 2 main dimensions: perceived susceptibility and perceived severity. The former refers to an individual’s subjective perception and/or beliefs about the personal risk of experiencing a health condition, whereas the latter refers to one’s beliefs regarding the seriousness of the health condition threatened [12,13].

**Figure 1:**
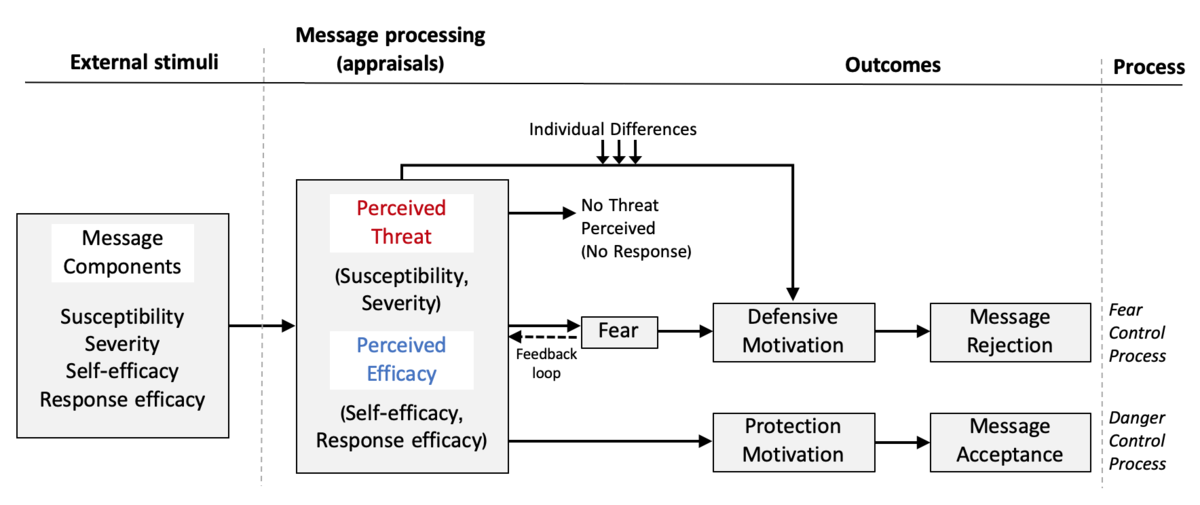
Extended Parallel Process Model, adapted from Witte.

When the perceived susceptibility and/or perceived severity is low (ie, *trivial or irrelevant*) [11], the recipient of the message will not be motivated to process the message, indicating that the efficacy will not be assessed and no response will take place to the fear appeal because the individual believes that the threat does not impact him or her and thus ignores it, as shown in Figure 1 [11,14]. However, when the processed threat message results in a moderate-to-high perceived threat, individuals become motivated and are likely to appraise efficacy (ie, an assessment of the efficacy of the recommended response) [11].

Perceived efficacy refers to the “cognition about the effectiveness, feasibility, and ease with which a recommended response alleviates or helps in avoiding a threat” [15]. The EPPM underlines 2 forms of efficacy under the perceived efficacy appraisal: perceived self-efficacy and perceived response efficacy. Perceived self-efficacy refers to one’s perception that he or she can execute the recommended behavior to avert the threat [15]. Perceived response efficacy refers to one’s belief about how effective the recommended response is in deterring the threat [15].

On the basis of the perceived efficacy level of the recommended behavior, individuals perform 1 of 2 responses: a danger control or a fear control response. If a message conveys both high threat and efficacy, it is predicted that it will lead individuals to control the danger, demonstrating protection motivation. Individuals will subsequently perform actions, typically the recommended message responses, to protect themselves against the threat. This means that the message succeeded in motivating people to take the recommended action (message acceptance). In contrast, when individuals perceive a high threat level but low efficacy in the message, they have a fear control response. Here, individuals will focus on alleviating the fear usually through psychological defense strategies (eg, defensive avoidance or denial) without taking action about the threat—a defense motivation outcome in the EPPM [14].

### Objectives

This study had 3 phases related to the objectives of this study: (1) design and develop a tailored message library based on the potentially different response states of the EPPM, (2) have clinical and research experts validate the content of the messages, and (3) have members of the target audience evaluate the perceived persuasiveness of the messages. Phase 1 of this study involved designing and developing the message library per the EPPM. In phase 2, the content validity of the developed message library was tested by research and clinical experts. The content validation prioritized 2 aspects: perceived relevance and clarity. In phase 3, a sample of patients with OSA assessed the perceived persuasiveness of the messages. The findings of this study will inform the development of mHealth interventions or information systems (IS) solutions targeting improved use of CPAP therapy among patients with OSA.

## Methods

### Phase 1: Developing the Message Library

According to the EPPM, an individual is most likely to act on a recommended response when he/she is in a danger control state. A message library is designed to motivate participants to move in that direction (ie, high efficacy and high threat). If an individual is already in a danger control state, then the messages are designed and strive to reinforce his/her state.

**Figure 2:**
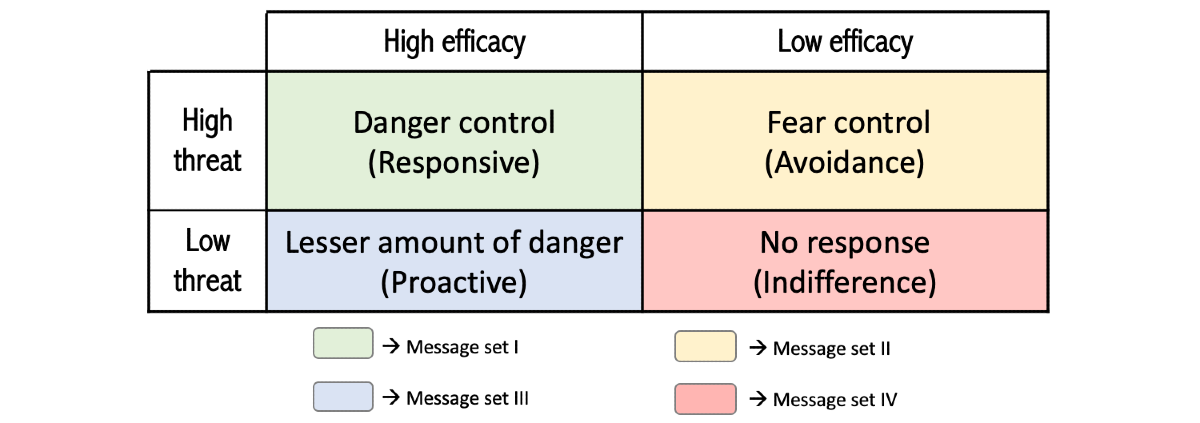
Extended Parallel Process Model potential response states and message types.

A total of 60 messages were selected from existing sources or designed to make the message content relevant based on behavioral beliefs per the EPPM. These messages can be categorized into 4 sets, 1 for each potential response state of the EPPM (ie, responsive, proactive, avoidance, and indifference), as shown in Figure 2 (Phase 2 provides further details). Each quadrant is titled with descriptors used by Rimal and Real [16]. Messages were designed to address both threat and efficacy components that need to be altered, reinforced, or initiated. In all 60 messages, the threat is the health consequence of untreated OSA, and the recommended response is using CPAP at least four hours per night (using CPAP ≥4 hours per night was generally admitted as the clinical and empirical level of use required for adherence [17]).

Message set I targets those who are in a responsive state. They were designed to reinforce existing perceptions of both threat and efficacy (eg, Untreated obstructive sleep apnea increases your risk of heart problems. Use your CPAP for better health).

Message set II targets those who are in the avoidance state. The messages were designed to recognize the threat, but mainly to increase the efficacy of using CPAP (eg, Try adjusting your mask pads and straps for a better mask fit. This may help reduce air leaks. Use your CPAP to sleep well and be well!).

Message set III targets those who are in a proactive state. It was designed to increase the threat and to reinforce existing perceptions of efficacy (eg, when left untreated, obstructive sleep apnea can lead to chronic diseases. Use your CPAP for better health!).

Message set IV targets those who are in the indifference state, in which they do not feel the threat of OSA and have doubts about the effectiveness of CPAP and their ability to use CPAP therapy. Thus, this set of messages was designed to initiate the efficacy, but mainly to convince patients of the severity and seriousness of untreated OSA (eg, Sleep apnea could be hurting your heart. Use the CPAP to sleep well and be well!) [18].

The initial library of 60 messages consisted of 21 text-based messages, 20 video-based messages, and 19 image-based messages. The rationale for developing 60 messages was that this would allow experts and patients to select the ones that are most relevant, clear, and persuasive. The best messages would then be used in an mHealth intervention that could be evaluated through an experiment.

Message generation was carried out in an iterative manner. First, a review of existing materials was conducted to collect available existing videos, infographics, and images that can be added to the library. For the selected ready-made videos and images, permission was obtained from the source to use the materials in the study. Participants were referred to the ready-made materials via the source’s web-based links. Text-, video-, and image-based messages were developed based on a review of the existing resources. The text, videos, and images provided in the messages were adapted from multiple sources.

Text messages were constructed by reviewing the following sources: the Mayo Clinic website [19], ResMed website [20,21], Breathe the Lung Association [22], the American Academy of Sleep Medicine [23], and the American Sleep Apnea Association [24]. The videos were YouTube-based videos provided through WebMD [25-27], the Cleveland Clinic [28,29], the American Academy of Sleep Medicine [30], Columbia Broadcasting System News [31], Lee Health [18], Johns Hopkins Medicine [32], and Mayo Clinic [33-35]. The length of the videos ranged from 43 seconds to approximately 5 min. Images and infographics were adopted from Adventist Health [36], National Jewish Health [37], Sleep Sherpa [38], the CPAP.com website [39], Sleep Well Respiratory Care [40], the American Sleep Apnea Association [41], Advanced Dental Sleep Treatment Center [42], Oral and Maxillofacial Surgeons [43], Very Well Health [44], Sleep Data [45], and Atlanta Headache temporomandibular joint Pain [46].

For the video- and image-based messages, the 12 principles of multimedia design outlined by Clark and Mayer [47] were used as a checklist to ensure that the messages address as many principles as possible. The length of the message was limited to no more than 160 characters to allow them to be delivered through a single mobile text message. The Gunning Fog test [48] was used to ensure that the text messages were at an eighth-grade reading level. The developed draft of the message library was maintained in a Microsoft Excel spreadsheet.

### Phase 2: Content Validation of the Messages by Experts

After the message library was developed, it was subjected to content validation by clinical and research experts. The content validation prioritized 2 aspects: the relevance of the messages to the theoretical concepts and the clarity of the messages. The experts’ suggestions were collected to improve the developed messages such that the content-validated messages could be tested by the target audience. The final message library was reviewed and improved based on the participants’ input. The messages were filtered by the content validity index for items (I-CVI), first for relevance and then for clarity.

#### Perceived Relevance

We first tested the relevance of the developed messages to the theoretical concepts (ie, perceived self-efficacy, perceived response efficacy, perceived severity, and perceived susceptibility) through expert evaluation. An expert is defined as an individual with acquired knowledge, experience, and/or skills in his/her field of expertise (eg, research and/or clinical practice) [49]. The messages were reviewed and validated by experts in the fields of pulmonology/sleep medicine, psychology, health promotion, and health IS and technology. The experts were board-certified pulmonologists in the field of sleep medicine and faculty or PhD candidates from Claremont Graduate University programs in behavioral and social sciences, health promotion sciences, and IS and technology. To participate, an expert had to meet at least one of the following criteria per the definition of *expert* stated earlier: (1) knowledge and experience in treating patients with sleep apnea, (2) experience in the use of theoretical concepts, (3) methodological knowledge in designing intervention studies, and/or (4) experience in research in chronic diseases management. The experts validated the messages by determining whether the messages reflected their underlying theoretical concepts. Drafts of all 60 messages were grouped into 6 subsets for validation (10 messages per subset). Each subset had a combination of modes: text, video, and image. We sent an email to potential experts inviting them to provide a rating and give comments using a web-based survey developed via Qualtrics (Qualtrics LLC). In particular, experts rated the relevance of each message to its underlying theoretical concepts using a 4-point ordinal scale: 1 (not relevant), 2 (somewhat relevant), 3 (quite relevant), and 4 (highly relevant).

#### Perceived Clarity

After the messages were checked for content relevance, the text- and image-based messages were further evaluated for clarity. The Qualtrics web-based survey asked experts to rate the clarity of each message using a 4-point ordinal scale: 1 (not clear), 2 (somewhat clear), 3 (quite clear), and 4 (highly clear). For each message, experts were asked to provide suggestions for improvement, if any. Overall, 4 message sets—2 text based and 2 image based—were created for validation. Video-based messages were not included in clarity validation because they are based on ready-made materials (YouTube links) provided by health organizations and were therefore assumed to have been already reviewed and validated by domain experts.

The first author attended 2 days of a scientific conference targeted at pulmonologists and sleep specialists held in October 2019 to recruit experts by asking them if they would participate in the study. Those who agreed to participate completed the survey on a laptop provided by the researcher.

### Phase 3: Message Testing by the Target Audience

After expert validation of the message library, the final version of the messages obtained from phase 2 was further validated by assessing the target audiences’ reaction to the messages’ persuasiveness. A cross-sectional survey was administered using a Qualtrics panel in February 2020. Qualtrics panels have been used extensively in health care research [50]. A Qualtrics panel is an opt-in research panel that offers participant recruitment services for researchers, where individuals who meet the study criteria are invited to participate. The Qualtrics-verified panel service provides access to potential participants who meet the study criteria. It uses third-party sources to verify the potential participants’ information and then sends a survey/study invitation to those who are eligible. This process is especially rigorous when potential participants indicate that they have been diagnosed with OSA. The survey’s technical functionality was tested by the first author before launching the survey.

**Figure 3:**
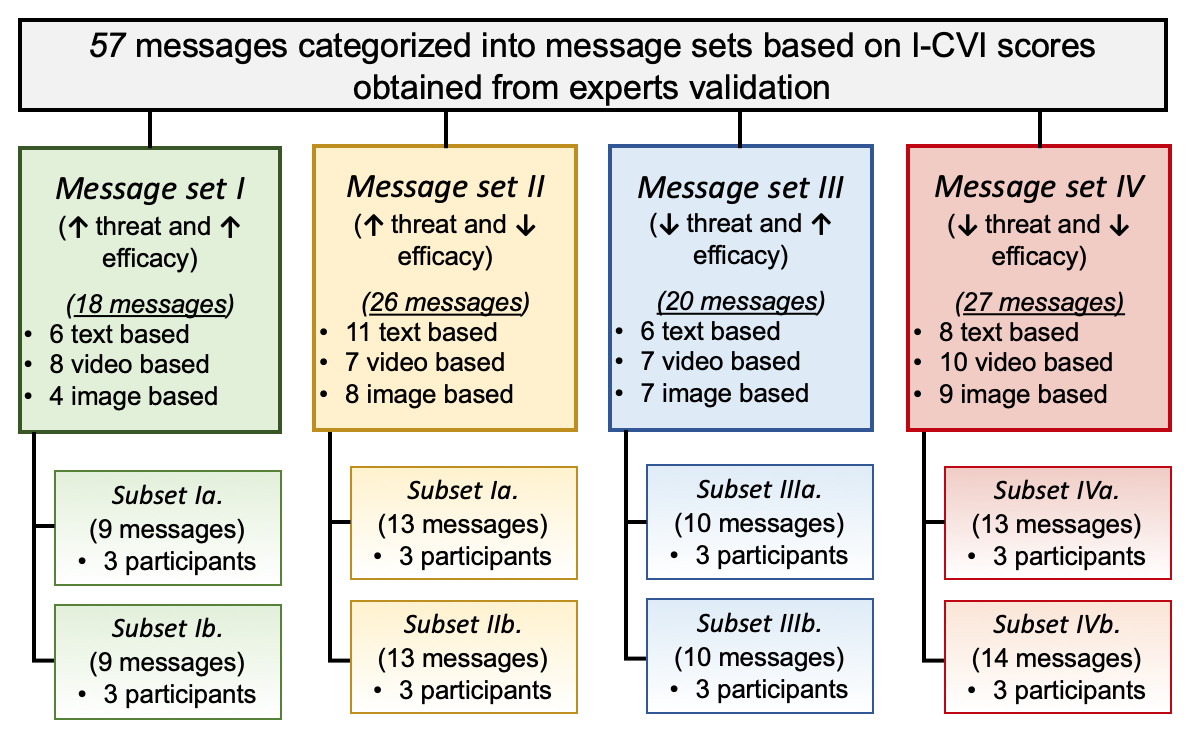
Message sets for perceived persuasiveness testing by patients with obstructive sleep apnea. Some messages were matched with multiple sets based on the obtained I-CVI score. I-CVI: content validity index for items.

Each of the 4 message sets that were obtained from phase 2, tailored based on whether participants have high or low efficacy and threat perceptions, were split into 2 surveys, creating 8 subsets (Figure 3). This was performed to shorten the length of the survey.

#### Participants

Web-based participants were recruited through a Qualtrics panel site (Qualtrics.com). Participants were a convenience sample of 24 adult patients with OSA across the United States who were selected based on their availability and willingness to participate in the study. Eligibility criteria included the following: (1) aged ≥18 years, (2) able to read and write in English, and (3) clinically diagnosed with OSA.

#### Procedure

The protocol for this study was approved by Claremont Graduate University’s institutional review board. The survey questionnaire was developed using Qualtrics’ web-based survey software. Recruitment was conducted by a Qualtrics project manager, where 676 potential participants in the United States, who indicated in their panel registration data that they have OSA, were invited via email to participate in the study. Qualtrics verified and confirmed their information. The invitation email communicated the opportunity to participate in an academic survey, approximate length, purpose, and a web link to the anonymous and confidential survey. To minimize participant response bias, the population of interest was not acknowledged (ie, participants were not told that they were invited because they had OSA). People who were interested in participating and who volunteered to do so were first required to read an introductory page that welcomed them and described the study and its goals. After the participants read the welcome page and study goals, they indicated their agreement on an electronic consent form. After signing the consent form, they were asked a few eligibility screening questions before they were cleared to participate in the study. Eligibility screening was added to recruit individuals in the target audience of an mHealth intervention or an IS solution (ie, adults diagnosed with OSA). The eligibility screening questions included a question about age and a question about the kinds of ailments that affects the participant. A total of 634 of the 676 potential participants did not meet the inclusion criteria; hence, they were excluded from the study. Those who met the eligibility screening criteria (N=42) were asked to report their demographic information, including ethnicity, educational level, and gender.

Next, they were asked to complete a survey to assess their efficacy and threat perceptions through the calculation of perceived self and response efficacy, perceived severity, and perceived susceptibility. Responses were then grouped into 4 quadrants as suggested by Popova [15]. To do this, the mean of each construct was calculated. Perceived efficacy was operationalized as the sum of the mean score of perceived self-efficacy and the mean score of perceived response efficacy as they are both considered as dimensions of the efficacy construct. Perceived threat was operationalized as the sum of the mean score of perceived severity and the mean score of perceived susceptibility as they are both considered as dimensions of the threat construct [11]. Sawyer and King [51] used the Self-Efficacy Measure for Sleep Apnea (SEMSA) with a score of 3 for each subscale as a critical indicator for tailoring an intervention. Thus, an average score of 3 was used as the critical indicator threshold to dichotomize the responses into high and low, with a score >3 being high and all others being classified as low. Thus, once the participants submitted their answers, a subset of messages from a set that matches their computed threat and efficacy perceptions appeared automatically on the screen, and they were asked to rate the extent to which each message is perceived persuasive on a 5-point Likert scale ranging from 1 (definitely not persuasive) to 5 (definitely persuasive). Once they were done, they hit the *submit* button.

Data were collected over 1 week from February 3 to 9, 2020. As monetary incentives have been found to be effective in enhancing the recruitment of participants [52], the participants were compensated upon the completion of the survey. Compensation was handled by Qualtrics. The average completion time for the survey was 25 min.

After obtaining all the participants’ responses, the last step in this phase was to determine the best 12 messages in each set. Subsequently, the messages were selected based on the obtained averages, and the best 12 messages were added to the final library.

#### Measures and Instrumentation

In all, 4 constructs were measured in phase 3: perceived self- and response efficacy, perceived severity, and perceived susceptibility. The constructs are self-reported multi-item scales drawn from previously validated measures.

##### Self-Efficacy Measure for Sleep Apnea

Perceived self-efficacy, perceived response efficacy, and perceived susceptibility were adapted from the SEMSA [53]. The SEMSA was developed based on Bandura’s social cognitive theory. It is divided into 3 subscales that measure self-efficacy, risk perception (perceived susceptibility), and outcome expectations (perceived response efficacy) [53]. The risk perception subscale is used for measuring perceived susceptibility as both terms refer to one’s perception of the likelihood that they will be affected by the health consequences of untreated OSA. For instance, Olsen [54] used SEMSA’s risk perception subscale to measure perceived susceptibility among patients clinically diagnosed with OSA. In addition, Witte et al [14] noted that the term *outcome expectations* in Bandura’s theory is *similar to response efficacy in fear appeals*. Therefore, the outcome expectations subscale was used to assess perceived response efficacy. Perceived self-efficacy, perceived response efficacy, and perceived susceptibility were assessed using a 4-point Likert scale ranging from 1 (not at all true) to 4 (very true). Higher scores denote greater perceived self-efficacy, greater perceived response efficacy, and higher perceived susceptibility.

##### Obstructive Sleep Apnea Knowledge and Beliefs

The OSA knowledge and beliefs (OSA-KAB) tool is a 16-item tool developed based on the health belief model to measure an individual’s knowledge and beliefs in the context of OSA [55]. The perceived severity subscale was adapted from the OSA-KAB tool [55]. Perceived severity was assessed using 3 items based on a 5-point Likert scale, ranging from 1 (strongly disagree) to 5 (strongly agree). Scores range from 3 to 15.

#### Attention Checks and Quality Screens

Attention checks were employed to confirm that survey respondents were reading the survey questions carefully and thoroughly. The responses were checked for validity by checking the participants’ answers to 2 embedded validity questions (ie, “please do not rate this statement so I know you are paying attention;” “please rate this statement at ‘not persuasive’ so I know you are paying attention”). One respondent failed one of the 2 embedded validity check questions, and 10 did not complete the entire survey; hence, they were excluded from the study.

Quality screens were also used to confirm that respondents spent an adequate amount of time completing the survey. The Qualtrics panel recommended excluding participants who completed the survey in one-third of the median time or less because that would indicate that the respondents were rushing through the survey. This criterion excluded 5 respondents.

### Analysis

#### Phase 2: Content Validation of the Messages by Experts

The I-CVI [56] was used to analyze the content validation for relevance and clarity. This allowed us to decide which messages to retain, delete, or adjust.

##### Perceived Relevance

Content validation of perceived relevance was conducted to ensure that each message included the correct theoretical concepts. The I-CVI was calculated by summing the messages that received scores of 3 or 4 and then dividing the sum by the total number of responses. Messages with I-CVIs ≤0.78 should be eliminated and/or reformulated [56]. However, Lynn [56] recommended that when there are ≤5 experts, an I-CVI of 1.00 must be obtained for the item/message to have content validity. If an expert rated a message with 1 or 2, he/she was asked to provide suggestions for improvement. The experts’ responses were analyzed and modifications were made to the first draft of the messages in accordance with the experts’ feedback.

To analyze the experts’ input, we first reviewed each expert’s feedback. Then, the experts’ ratings and suggestions were transcribed into a table in a Microsoft Excel sheet. On the basis of the ratings of each message, the I-CVI was calculated to determine the extent to which each message related to each of the theoretical concepts. We then categorized each message into message sets based on the I-CVI scores. Some messages can be associated with more than one set. Specifically, we categorized each message dependent on whether an I-CVI of 1.00 was addressed to 0, 1, or 2 elements of threat and efficacy. As message set I targets those who are in the responsive state (high threat and high efficacy), it is designed to reinforce existing perceptions of both threat and efficacy; therefore, messages that received an I-CVI of 1.00 for at least one element of threat and at least one element of efficacy were matched to message set I. As message set II targets those who are in the avoidance state (high threat and low efficacy), the messages are designed mainly to increase efficacy. Thus, messages that received an I-CVI of 1.00 for at least one element of efficacy were matched to set II. As message set III targets those who are in the proactive state (low threat and high efficacy), it is designed to increase the threat and to reinforce existing perceptions of efficacy. Thus, messages that received an I-CVI of 1.00 for 1 or 2 elements of threat and 1 element of efficacy were matched to message set III. As message set IV targets those who are in the indifference state (low threat and low efficacy), it is designed to initiate the efficacy and mainly to convince patients of the threat of untreated OSA. Thus, messages with an I-CVI of 1.00 for at least one element of threat and at least one element of efficacy or 1 or 2 elements of threat were matched to set IV.

##### Perceived Clarity

The I-CVI was computed as the number of experts giving a rating of 3 (quite clear) or 4 (*highly clear*) divided by the number of experts. An I-CVI score of 1.00 denotes a 100% agreement among the experts. The experts’ suggestions for improvement were also analyzed. Messages with low I-CVI scores (ie, <1.00) for clarity were reformulated based on the experts’ written comments or deleted.

#### Phase 3: Message Testing by the Target Audience

Descriptive statistics were computed using frequency counts and percentages for categorical variables. To determine the best 12 messages in each set, participants’ responses in each subset were analyzed as follows. Patient feedback on the survey was downloaded in Microsoft Excel. Each response state group (ie, responsive, proactive, avoidance, and indifference) was presented in a separate worksheet. The perceived persuasiveness ratings were provided next to each message and the average score of the ratings was calculated for each message. Then, the messages were sorted from highest to lowest based on the average score. This information enabled us to select the 6 best messages from each subset, yielding 12 from each message set.

## Results

### Phase 2: Content Validation of the Messages by Experts

#### Perceived Relevance

A total of 18 experts completed the survey, 3 for each subset (Table 1 provides the experts’ characteristics). In the first draft of 60 messages, 1 message was eliminated (“Experiencing a return of symptoms or feeling like the CPAP is no longer working? Your mask may have an air leak”) because of its low I-CVI score (<1.00) on each of the 4 theoretical concepts and because of an expert’s comment that “there are acceptable leaks.” As a result, the second draft included 59 messages.

The message library consisted of the following: 18 messages in set I (high threat and high efficacy: 6 text based, 4 image based, and 8 video based), 26 messages in set II (high threat and low efficacy: 11 text based, 8 image based, and 7 video based), 20 messages in set III (low threat and high efficacy: 6 text based, 7 image based, and 7 video based), and 27 messages in set IV (low threat and low efficacy: 8 text based, 9 image based, and 10 video based). It is important to note that some messages were matched with multiple sets based on the obtained I-CVI score of the messages’ perceived relevance to efficacy and threat constructs.

**Table 1:** Experts’ characteristics for message relevance validation.

Characteristics		Message subset 1	Message subset 2	Message subset 3	Message subset 4	Message subset 5	Message subset 6
Experts, n **(%)**		3 (16.6)	3 (16.6)	3 (16.6)	3 (16.6)	3 (16.6)	3 (16.6)
**Gender, n (%)**							
	Female	2 (67)	0 (0)	1 (33)	2 (67)	2 (67)	2 (67)
	Male	1 (33)	3 (100)	2 (67)	1 (33)	1 (33)	1 (33)
**Area of expertise, n**							
	Pulmonology and sleep medicine	0	0	1	1	0	0
	BSS^a^	1	0	2	0	1	1
	IST^b^	1	2	0	1	0	1
	HPS^c^	1	1	0	1	2	1
**Current position, n**							
	Physician	0	0	1	1	0	0
	Professor	1	1	1	0	1	1
	Psychologist	0	0	1	0	0	0
	PhD candidate	2	2	0	2	2	2

^a^BSS: behavioral and social science.

^b^IST: information systems and technology.

^c^HPS: health promotion science.

#### Perceived Clarity

A total of 16 experts completed the survey at the conference, and 3 experts provided their email addresses so that we could send them the survey link and they could complete the survey during their free time. As a result, a convenience sample of 19 experts (N=19) was used for clarity content validation. The experts were physicians and faculty members with expertise in the fields of pulmonology and/or sleep medicine.

The text-based messages were grouped into 2 sets for validation, and 3 experts evaluated the clarity of each set except for 1 set. That 1 set was evaluated by 4 experts as 1 expert had zero expertise in the field of pulmonology and/or sleep medicine. Then, we analyzed the data, and 8 messages (8/20) received an I-CVI for clarity <1.00. Modifications based on the experts’ comments were made to these messages. We also modified 3 messages that received an I-CVI of 1.00 to address the experts’ comments (eg, For the message “Do you feel sleepy or tired in the morning? Poor sleep may be a reason for sleepiness or tiredness. Use CPAP for at least 4 hours a night to feel better!” one expert commented, “Not only in the morning, probably just mention during the day”). As a result, 11 messages were reworded based on the experts’ suggestions. We then created a survey to evaluate the clarity of the reworded messages. A total of 4 experts who were recruited from the conference completed the survey. The analysis of the reworded messages showed that 10 out of 11 messages received an I-CVI of 1.00 and 1 received an I-CVI of 0.75. However, 3 messages that received an I-CVI of 1.00 and 1 message that received an I-CVI of 0.75 were modified to address the experts’ comments.

The image-based messages were also grouped into 2 sets for validation. Overall, 6 experts completed the survey (3 for each set of messages). Of the 19 image-based messages, 2 were eliminated because they received an I-CVI <1.00 as well as critical comments. In particular, 1 of the experts commented that 1 of the messages was “too wordy” and that the other message included inaccurate information (ie, the message should have indicated that the CPAP pumps air, not oxygen). Two messages were modified to address the experts’ comments (eg, “the background color is too dark” and “perhaps changing the white font to black”).

Finally, the modified messages were further tested for clarity by 3 additional experts. The modified messages consisted of 4 text-based messages that were modified based on the experts’ comments and 2 image-based messages. Thus, 6 messages were included in *set 6* for clarity validation. The results show that all 6 messages received an I-CVI of 1.00. Therefore, a library of 57 messages (20 text-, 17 image-, and 20 video-based messages) was ready for use in the patient validation phase.

### Phase 3: Message Testing by the Target Audience

#### Participants

**Figure 4:**
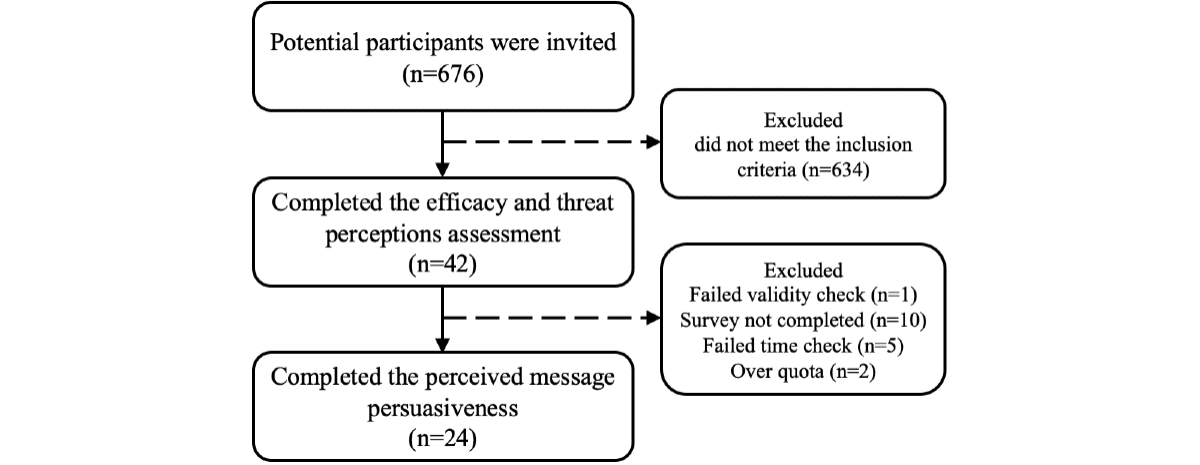
Patients with obstructive sleep apnea message testing process.

A total of 676 potential participants were invited to participate in the web-based survey, 42 met the study criteria, and 30 participants completed the survey, of which 6 were excluded for not meeting the time quality screens or failed 1 of the 2 embedded validity questions (Figure 4).

Overall, 24 participants completed the web-based survey (7 men and 17 women). The average age of the participants was between 45 and 55 years. The demographic characteristics of the participants are presented in Table 2. Overall, the participants (N=24) were predominantly women (n=17), white (n=22), and reported having a college degree (n=12).

Participants were categorized into 4 groups based on whether they had high or low efficacy and threat perceptions (ie, responsive, avoidance, proactive, and indifference). As stated earlier, an average score of 3 was used as a critical indicator threshold to dichotomize the responses into high and low following Sawyer et al [51], with a score >3 being high and all others being classified as low. The indifference group had all women, only the responsive group had nonwhites, and only the proactive group had participants who were aged 65 years.

**Table 2:** Demographics of participants by response state.

Demographics^a^		Responsive (high threat and high efficacy)	Avoidance (high threat and low efficacy)	Proactive (low threat and high efficacy)	Indifference (low threat and low efficacy)	Combined
Participants, n (%)		6 (25)	6 (25)	6 (25)	6 (25)	24 (100)
**Age (years), n (%)**						
	18-24	0 (0)	0 (0)	0 (0)	2 (33)	2 (8)
	25-34	2 (33)	2 (33)	0 (0)	0 (0)	4 (17)
	35-44	1 (17)	2 (33)	0 (0)	0 (0)	3 (13)
	45-54	1 (17)	1 (17)	3 (50)	2 (33)	7 (29)
	55-64	2 (33)	1 (17)	0 (0)	2 (33)	5 (21)
	65-74	0 (0)	0 (0)	2 (33)	0 (0)	2 (8)
	75-84	0 (0)	0 (0)	1 (17)	0 (0)	1 (4)
**Gender, n (%)**						
	Female	4 (67)	3 (50)	4 (67)	6 (100)	17 (71)
	Male	2 (33)	3 (50)	2 (33)	0 (0)	7 (29)
**Ethnicity, n (%)**						
	White	4 (66)	6 (100)	6 (100)	6 (100)	22 (92)
	Black	1 (17)	0 (0)	0 (0)	0 (0)	1 (4)
	Hispanic	1 (17)	0 (0)	0 (0)	0 (0)	1 (4)
**Education, n (%)**						
	High school	2 (33)	3 (50)	3 (50)	2 (33)	10 (42)
	College	3 (50)	3 (50)	3 (50)	3 (50)	12 (50)
	Master	0 (0)	0 (0)	0 (0)	1 (17)	1 (4)
	Doctorate	1 (17)	0 (0)	0 (0)	0 (0)	1 (4)

^a^Response state refers to participants’ categorization based on whether they have high/low threat and efficacy perceptions.

#### Message Set I

The message set I targets those who were in the responsive state (high threat and high efficacy), in which messages were designed to reinforce the patient’s existing threat perception of untreated OSA and efficacy perception of using the CPAP device.

For set Ia, 7 messages received an average score of 4.67, 1 message had a mean score of 4.33, and 1 message had a mean score of 4.00. We intended to select the top 6 messages, sorted from highest to lowest based on the average score from each subset (ie, messages with the top high average scores), yielding 12 for set I. As 7 messages received an average score of 4.67, which represents the highest average score in this subset, a random number generator was used to select 6 of the 7 messages to be used in the final set. This was done to meet the predetermined message quantity.

For set Ib, 1 message received a mean score of 5.00, 1 message had a mean score of 4.67, 2 messages had a mean score of 4.33, and 3 messages had a mean score of 4.00. Among those that received a mean score of 4.00, 2 messages were randomly selected to be included in the final set to meet the predetermined message quantity. As a result, 12 messages were selected for set I, including 3 image-based, 6 video-based, and 3 text-based messages. Among the 18 messages that were under consideration, 12 messages were rated higher in perceived persuasiveness by patients with OSA who were in the responsive state and thus were added to the final library. Multimedia Appendix 1 provides the final 12 messages.

#### Message Set II

This message set targets those who were in the avoidance state (high threat and low efficacy), in which messages were designed mainly to increase patients’ efficacy perception of using their CPAP device.

For set IIa, 2 messages received a mean score of 4.33 and 6 messages received a mean score of 4.00. Among the 6 messages that received a mean score of 4.00, 4 messages were selected randomly to be included in the final set II.

For set IIb, 1 message received a mean score of 4.67, 3 messages received a mean score of 4.33, and 3 messages received a mean score of 4.00. Among these messages with a mean score of 4.00, 2 messages were randomly selected to be included in the final set. Among the 26 messages that were under consideration, the selected 12 messages were perceived to be more persuasive than other messages in the set based on the feedback gained from patients with OSA who were in the avoidance state. As a result, 12 messages were added to the final message set II, including 6 image-based, 3 video-based, and 3 text-based messages (Multimedia Appendix 1).

#### Message Set III

This message set targets those who were in the proactive state (low threat and high efficacy), in which messages were designed to increase patient’s threat perception of untreated OSA and reinforce existing efficacy perception of using their CPAP device.

For set IIIa, 1 message received a mean score of 5.00 and 5 messages received a mean score of 4.67, yielding 6 messages to be added to the final set. For set IIIb, 3 messages received a mean score of 4.67 and 6 messages received a mean score of 4.33. Three of the 6 messages that received a mean score of 4.33 were selected randomly to be included in the final set. As a result, 12 messages were included in the final set, including 3 image-based, 5 video-based, and 4 text-based messages (Multimedia Appendix 1).

#### Message Set IV

This message set targets those who were in the indifference state (low threat and low efficacy), in which messages were designed to initiate the patient’s efficacy of using their CPAP device and to convince patients of the threat of untreated OSA.

For message set IVa, 2 messages received a mean score of 4.33 and 6 messages received a mean score of 4.00; thus 4 messages were selected randomly to be added to the final set. For message set IVb, 1 message received a mean score of 4.67, 4 messages received a mean score of 4.33, and 3 messages had a mean score of 4. One message was randomly selected from the 3 messages with a mean score of 4.00 and was added to the final set. As a result, 12 messages were selected for the final set IV, including 4 image-based, 4 video-based, and 4 text-based messages (Multimedia Appendix 1).

## Discussion

This study aimed to develop, validate the content of, and test a set of messages for an mHealth behavior change support system that will target patients with OSA who are noncompliant with their CPAP therapy. The goal was to motivate them to use their CPAP device and become compliant with their CPAP therapy. The multiphase message development, content validation, and testing phases described herein offer a practical guide for researchers and health care practitioners to use for tailored motivational message development. The tailored messages were built based on the EPPM targeting patients with OSA to motivate them to use their CPAP devices.

In phase 1, messages were derived from the literature and a review of the existing materials. This phase was made easier due to the availability of appropriate videos and infographics; developing image- and video-based messages from scratch can be costly and time-consuming. Fertman and Allensworth [57] noted the importance of reviewing existing materials that can assist the researcher or practitioner in deciding whether to use them as they are, modify them, or develop new messages from scratch.

In phase 2, the enumerated content validity of the messages was established through the utilization of I-CVI [56]. A total of 57 messages were found to have acceptable content relevance and clarity. The experts’ content testing and their comments enriched and improved the messages in terms of clarity and relevance to the theoretical concepts. The suggestions for modification of some sentences and/or the presentation of information provided guidance to develop and design messages. The expert ratings of the messages assisted us in the process of categorizing messages into high or low threat and efficacy perception. The value of expert knowledge and feedback has been recognized in message design and content validation [58-60].

In phase 3, the messages were further tested using a convenience sample of the target audience for perceived persuasiveness. Pretesting potential messages to identify the ones that are more likely to be relatively effective is an important step to assist the message designer or researcher in the process of selecting messages to include in the intervention [61].

Acquiring feedback from the target audience and experts in the subject matter assisted us in revising and selecting a set of messages that was presumed to be more effective. The results showed that each of the 4 final message sets included all 3 modes (image-, video-, and text-based messages). Studies note the advantages of video-based messages that require less mental and cognitive effort compared with plain text messages, freeing resources to process the main messages, thus making them potentially more applicable for those with low educational levels [62,63]. However, text-based and image-based messages may be more applicable in situations such as being in a noisy place or having a limited amount of time. As a result, we believe that one size does not fit all. Having a variety of modes is advantageous for reaching and considering as many different individual characteristics and/or preferences as possible as well as the range of environmental situations that someone may experience in daily life.

The main contribution of this study was the development of a selection process for validating the content of and testing a set of messages to be used in an mHealth behavior change support system solution in the context of OSA. There is a need for future intervention-oriented research using these messages to examine and determine their impact on patients’ short- and long-term CPAP usage. Future research should evaluate IS-/information technology–based solution features and strategies for implementation in delivering and presenting these messages as a means to support the motivation of noncompliant patients with OSA. It will also be important to assess the effectiveness of these features and strategies in promoting CPAP usage among patients with OSA. If the intervention demonstrates effectiveness, it could prove to be an efficient motivational tool for CPAP users. It can be delivered to a large number of patients with OSA.

### Conclusions

Poor use of CPAP therapy among patients with OSA is a serious problem. Effective interventions are needed to improve treatment usage by patients with chronic health conditions such as OSA to enhance health outcomes and quality of life [64]. This study was designed to help promote CPAP usage among patients with OSA. This was a formative study for developing and validating the content of a set of messages to be used in an mHealth sleep behavior change support system.

After the process of content validation and testing, the final library of messages met the criteria for clarity, relevance, and persuasiveness. This study emphasizes the importance of developing and validating the content of motivational messages, grounded in theory, across the 4 possible response states in terms of high/low efficacy and threat perceptions. We expect the potential impact of these message sets will come from serving as a resource for different IS solutions or mHealth interventions targeted at increasing CPAP usage/adherence among patients with OSA. Through the evaluation of these IS solutions or mHealth interventions, more insights will be gained on the efficacy and effectiveness of messages on CPAP adherence.

This study has some limitations. First, there is a selection bias for the experts’ responses in phase 2. All experts had clinical and/or research expertise, but most of those who participated in relevance content validation were research experts (ie, either professors or PhD candidates) from different disciplines. However, most of the experts who participated in the clarity content validation were clinical experts in the field of pulmonology and/or sleep medicine. As the experts were derived from different disciplines, and each message set was evaluated by different experts (3 experts evaluated each set), there is a potential for selection bias. Second, the majority of patients with OSA who participated in the perceived persuasiveness testing (phase 3) were white and women. Finally, the small sample size is based on a convenience sampling technique. Having a small sample size limited the variety of statistical techniques that could be used. However, this study was not intended to generalize the results to a larger population; thus, considering the available resources, using convenience purposeful sampling was applicable in this case. Purposively enrolling participants will encompass a range of perspectives and views regarding the constructed messages.

## Appendix

Multimedia Appendix 1 Final Message Library.

## Abbreviations

CPAP: continuous positive airway pressure
EPPM: extended parallel process model
I-CVI: content validity index for items
IS: information systems
mHealth: mobile health
OSA: obstructive sleep apnea
OSA-KAB: obstructive sleep apnea knowledge and beliefs
SEMSA: Self-Efficacy Measure for Sleep Apnea

## References

[1] Lattimore Jl, Celermajer DS, Wilcox I (2003). Obstructive sleep apnea and cardiovascular disease. J Am Coll Cardiol.

[2] An E (2020). Which Came First, Obstructive Sleep Apnea or Hypertension? A Retrospective Study of Electronic Records Over 10 Years, With Separation by Sex. medRxiv.

[3] Benjafield AV, Ayas NT, Eastwood PR, Heinzer R, Ip MS, Morrell MJ, Nunez CM, Patel SR, Penzel T, Pépin J, Peppard PE, Sinha S, Tufik S, Valentine K, Malhotra A (2019). Estimation of the global prevalence and burden of obstructive sleep apnoea: a literature-based analysis. Lancet Respiratory Med.

[4] de Luca CG, Pachêco-Pereira C, Aydinoz S, Major PW, Flores-Mir C, Gozal D (2015). Biomarkers associated with obstructive sleep apnea and morbidities: a scoping review. Sleep Med.

[5] Wickwire EM, Albrecht JS, Towe MM, Abariga SA, Diaz-Abad M, Shipper AG, Cooper LM, Assefa SZ, Tom SE, Scharf SM (2019). The impact of treatments for OSA on monetized health economic outcomes: a systematic review. Chest.

[6] Weaver TE, Sawyer AM (2010). Adherence to continuous positive airway pressure treatment for obstructive sleep apnoea: implications for future interventions. Indian J Med Res.

[7] Olsen S, Smith SS, Oei TP, Douglas J (2012). Motivational interviewing (MINT) improves continuous positive airway pressure (CPAP) acceptance and adherence: a randomized controlled trial. J Consult Clin Psychol.

[8] Weaver TE (2019). Novel aspects of CPAP treatment and interventions to improve CPAP adherence. J Clin Med.

[9] Sawyer AM, Gooneratne NS, Marcus CL, Ofer D, Richards KC, Weaver TE (2011). A systematic review of CPAP adherence across age groups: clinical and empiric insights for developing CPAP adherence interventions. Sleep Med Rev.

[10] Kendzerska T, Wilton K, Bahar R, Ryan CM (2019). Short- and long-term continuous positive airway pressure usage in the post-stroke population with obstructive sleep apnea. Sleep Breath.

[11] Witte K (1992). Putting the fear back into fear appeals: the extended parallel process model. Commun Monogr.

[12] Maloney EK, Lapinski MK, Witte W (2011). Fear appeals and persuasion: A review and update of the Extended Parallel Process Model. Soc Personal Psychol.

[13] Gore TD, Bracken CC (2005). Testing the theoretical design of a health risk message: reexamining the major tenets of the extended parallel process model. Health Educ Behav.

[14] Witte K, Meyer G, Martell D (2001). Effective Health Risk Messages: A Step-By-Step Guide.

[15] Popova L (2012). The extended parallel process model: illuminating the gaps in research. Health Educ Behav.

[16] Rimal RN (2003). Perceived risk and efficacy beliefs as motivators of change: use of the risk perception attitude (RPA) framework to understand health behaviors. Hum Commun Res.

[17] Haba-Rubio J, Vujica J, Franc Y, Michel P, Heinzer R (2019). Effect of CPAP treatment of sleep apnea on clinical prognosis after ischemic stroke: an observational study. J Clin Sleep Med.

[18] Lee Health (2015). Sleep Apnea Could be Hurting Your Heart. YouTube.

[19] (2020). Sleep Apnea - Symptoms And Causes. Mayo Clinic.

[20] (2020). Living With CPAP: 7 Tips for a Better Experience. ResMed.

[21] (2020). CPAP Therapy. ResMed.

[22] (2018). Sleep Apnea. Lung Cancer Canada.

[23] (2018). Risk of Motor Vehicle Accidents is Higher in People With Sleep Apnea. The American Academy of Sleep Medicine (AASM).

[24] (2020). Troubleshooting Guide for CPAP Problem. American Sleep Apnea Association (ASAA).

[25] 3 Tips for Sleeping with a CPAP. YouTube.

[26] What Cause Sleep Apnea?. YouTube.

[27] How to Clean Your CPAP. YouTube.

[28] Cleveland C PAP Therapy: What to Expect. YouTube.

[29] Harneet Walia, M.D. - 'Study: Common Sleep Apnea Treatment Reduces Drowsy Driving'. YouTube.

[30] Sleep Apnea-Sleep Education. YouTube.

[31] Sleep Apnea Increase Risk of Heart Attack, Study Finds. YouTube.

[32] Obstructive Sleep Apnea. YouTube.

[33] Obstructive Sleep Apnea. YouTube.

[34] Mayo Clinic Minute: Signs of a Sleep Disorder. YouTube.

[35] Sleep Well with Positive Airway Pressure (PAP). YouTube.

[36] How Sleep Disorders Affect Your Heart. Adventist Health.

[37] Top 10 CPAP Life Hacks. National Jewish Health.

[38] Sleep Apnea. Everything Birds.

[39] Replacement Schedule. CPAP.

[40] What is Obstructive Sleep Apnea?. WordPress.

[41] How to Survive CPAP During Allergy Season. American Sleep Apnea Association.

[42] Hypertension Affects Your Whole Body. Omaha Sleep Apnea & Snoring Dentist: Dr Roger Roubal.

[43] Obstructive Sleep Apnea. AAOMS: Experts in Face, Mouth & Jaw Surgery.

[44] Risks of Untreated Sleep Apnea. Verywell Health - Know More. Feel Better.

[45] Prevalence of Sleep Apnea in Associated Co-Morbidities. Squarespace.

[46] Untreated Obstructive Sleep Apnea has Negative Effects on Medical Conditions. Craniofacial Pain and Dental Sleep Center of Georgia.

[47] Clark RC, Mayer RE (2016). E-Learning and the Science of Instruction: Proven Guidelines for Consumers and Designers of Multimedia Learning.

[48] Gunning R (2016). The fog index after twenty years. Int J Bus Commun.

[49] Dey S, Sedera D (2011). Information System Experts: Definition, Identification, and Application. Proceedings of the 19th European Conference on Information Systems.

[50] Williams J, Hadjistavropoulos T, Ghandehari OO, Malloy DC, Hunter PV, Martin RR (2016). Resilience and organisational empowerment among long-term care nurses: effects on patient care and absenteeism. J Nurs Manag.

[51] Sawyer AM, King TS, Weaver TE, Sawyer DA, Varrasse M, Franks J, Watach A, Kolanowski AM, Richards KC (2019). A tailored intervention for PAP sdherence: the SCIP-PA trial. Behav Sleep Med.

[52] Grady C (2005). Payment of clinical research subjects. J Clin Invest.

[53] Weaver TE, Maislin G, Dinges DF, Younger J, Cantor C, McCloskey S, Pack AI (2003). Self-efficacy in sleep apnea: instrument development and patient perceptions of obstructive sleep apnea risk, treatment benefit, and volition to use continuous positive airway pressure. Sleep.

[54] Olsen S, Smith S, Oei T, Douglas J (2008). Health belief model predicts adherence to CPAP before experience with CPAP. Eur Respir J.

[55] Homoud M (2019). Adults’ Knowledge and Beliefs Surrounding Obstructive Sleep Apnea. Seton Hall University.

[56] Lynn MR (1986). Determination and quantification of content validity. Nurs Res.

[57] Fertman CI, Allensworth DD (2016). Health Promotion Programs: From Theory to Practice.

[58] Lima I, Galvão M, Pedrosa SC, Silva CA, Pereira ML (2017). Validação de mensagens telefônicas para promoção da saúde de pessoas com HIV. Acta Paul Enferm.

[59] Helitzer D, Hollis C, Cotner J, Oestreicher N (2009). Health literacy demands of written health information materials: an assessment of cervical cancer prevention materials. Cancer Control.

[60] Celik S, Cosansu G, Erdogan S, Kahraman A, Isik S, Bayrak G, Bektas B, Olgun N (2015). Using mobile phone text messages to improve insulin injection technique and glycaemic control in patients with diabetes mellitus: a multi-centre study in Turkey. J Clin Nurs.

[61] O’Keefe DJ (2019). Message pretesting using perceived persuasiveness measures: reconsidering the correlational evidence. Commun Methods Meas.

[62] Stanczyk NE, Smit ES, Schulz DN, de Vries H, Bolman C, Muris JW, Evers SM (2014). An economic evaluation of a video- and text-based computer-tailored intervention for smoking cessation: a cost-effectiveness and cost-utility analysis of a randomized controlled trial. PLoS One.

[63] Walthouwer MJ, Oenema A, Soetens K, Lechner L, De Vries H (2013). Systematic development of a text-driven and a video-driven web-based computer-tailored obesity prevention intervention. BMC Public Health.

[64] Long H, Bartlett YK, Farmer AJ, French DP (2019). Identifying brief message content for interventions delivered via mobile devices to improve medication adherence in people with type 2 diabetes mellitus: a rapid systematic review. J Med Internet Res.

