# Supplementary material for: A Tailored Motivational Messages Library for a Mobile Health Sleep Behavior Change Support System to Promote Continuous Positive Airway Pressure Use Among Patients With Obstructive Sleep Apnea: Development, Content Validation, and Testing
Source: JMIR Mhealth Uhealth. 2020 Aug 12;8(8):e18793. doi: 10.2196/18793 (PMC7450383; doi:10.2196/18793)
Supplement: Multimedia Appendix 1 [file mhealth_v8i8e18793_app1.docx]

| **Message** | **Mode** | **Content** |
| --- | --- | --- |
| Set1_Msg1 | Image-based | Here are some symptoms of sleep apnea. You can reduce them by using the CPAP at least 4 hours a night! https://tinyurl.com/y52e34m7 |
| Set1_Msg2 | Video-based | Your health is your wealth. Watch this video to learn more about sleep apnea: https://www.youtube.com/embed/VPwpJd8aBzM |
| Set1_Msg3 | Text-based | Untreated obstructive sleep apnea increases your risk of high blood pressure. Use your CPAP for better health! |
| Set1_Msg4 | Image-based | Learn about how sleep disorders affect your heart: <https://www.adventisthealth.org/images/import/wp-content/uploads/2016/03/sleep-disorders-infographic.png> |
| Set1_Msg5 | Video-based | There's nothing like a good night's sleep. Find out how CPAP changed Todd’s life in this 2-min video: https://www.youtube.com/embed/z12MEPiG4cg |
| Set1_Msg6 | Text-based | Untreated obstructive sleep apnea increases your risk of heart problems. Use your CPAP for better health! |
| Set1_Msg7 | Video-based | Having some problems using the CPAP machine? You are not alone! Watch this video for some CPAP tips:  <https://www.youtube.com/embed/tgSMlsoNDFg> |
| Set1_Msg8 | Text-based | Sleep apnea can increase the risk of motor vehicle and workplace accidents. Using a CPAP device for at least 4 hours a night can help you to reduce this risk! |
| Set1_Msg9 | Image-based | 13. Don’t wait. Act now: <https://pbs.twimg.com/media/DEksFuGVoAA8R9p.jpg> |
| Set1_Msg10 | Video-based | Untreated sleep apnea increases the risk of having a heart attack. Use your CPAP for better health! Watch this video: https://www.youtube.com/embed/vbTCWLnoK2A |
| Set1_Msg11 | Video-based | Sleep apnea could be hurting your heart. Use the CPAP to sleep well and be well! https://www.youtube.com/embed/vSxEvWjpqBo |
| Set1_Msg12 | Video-based | Obstructive sleep apnea can make you feel tired all the time. Find out more in this video: https://www.youtube.com/embed/6bqTOfwvWhk |

**Final Message Library**

***Set I final messages***

| **Message** | **Mode** | **Content** |
| --- | --- | --- |
| Set2_Msg1 | Video-based | Follow these 3 tips for a smooth transition to fall asleep while wearing a CPAP mask. Watch this video:  https://www.youtube.com/embed/GzrJCqHCx8U |
| Set2_Msg2 | Image-based | Here are some tips on how to use the CPAP during allergy season:  <https://www.sleepapnea.org/wp-content/uploads/2017/04/CPAP-and-Allergies-ASAA-infoG.png> |
| Set2_Msg3 | Text-based | Do you feel tired even after a full night of sleep? Using your CPAP device for at least 4 hours a night can help you to solve this problem. |
| Set2_Msg4 | Image-based | Here are some useful tips for you:  <https://cgu.co1.qualtrics.com/CP/Graphic.php?IM=IM_cGY1VNtzzVI26AR> |
| Set2_Msg5 | Text-based | The more you use CPAP while sleeping, the better you may feel. You can do it. Sleep well! |
| Set2_Msg6 | Image-based | Some CPAP problems and how to solve them:  <https://cgu.co1.qualtrics.com/CP/Graphic.php?IM=IM_a8Aetk1iOgVhusd> |
| Set2_Msg7 | Image-based | Get to know your CPAP machine!  https://5faacd0c97cf0d23c6dc-064f3cd61bcd6e809053bf2fe3432ece.ssl.cf1.rackcdn.com/replacementschedulecpapdotcom.pdf |
| Set2_Msg8 | Video-based | Having some problems using the CPAP machine? You are not alone! Watch this video for some CPAP tips:  <https://www.youtube.com/embed/tgSMlsoNDFg> |
| Set2_Msg9 | Video-based | Watch this video for some tips on how to clean your CPAP:  https://www.youtube.com/embed/PgUeDkZvS4M |
| Set2_Msg10 | Image-based | Here are some CPAP life hacks:  https://www.nationaljewish.org/getattachment/Health-Insights/health-infographics/top-10-cpap-life-hacks/Top-10-CPAP-Life-Hacks-(1).jpg.aspx |
| Set2_Msg11 | Text-based | Try adjusting your mask pads and straps for a better mask fit. This may help reduce air leaks. Use your CPAP to sleep well and be well! |
| Set2_Msg12 | Image-based | Here are some tips for CPAP mask problems:  <https://cgu.co1.qualtrics.com/CP/Graphic.php?IM=IM_bmiulN4eHc6LW3b> |

***Set II final messages***

***Set III final messages***

| **Message** | **Mode** | **Content** |
| --- | --- | --- |
| Set3_Msg1 | Text-based | Sleep apnea can increase the risk of motor vehicle and workplace accidents. Using a CPAP device for at least 4 hours a night can help you to reduce this risk! |
| Set3_Msg2 | Image-based | Learn how sleep apnea affects your whole body!  <https://www.whywesnore.com/x/lc-content/uploads/2017/02/2016-sleep-apnea-infographic-8-2.png> |
| Set3_Msg3 | Video-based | Watch this video to learn more about sleep apnea:  <https://www.youtube.com/embed/lu-pMXDZ8IA> |
| Set3_Msg4 | Text-based | Untreated obstructive sleep apnea increases your risk of high blood pressure. Use your CPAP for better health! |
| Set3_Msg5 | Video-based | Want to learn more about sleep apnea and the benefits of using CPAP? Watch this video: <https://www.youtube.com/embed/8_6CDuSiajA> |
| Set3_Msg6 | Text-based | When left untreated, obstructive sleep apnea can lead to chronic diseases. Use your CPAP for better health. |
| Set3_Msg7 | Image-based | Learn about the risks of untreated sleep apnea:  <https://myoms.org/wp-content/uploads/2019/09/09_obstructive_sleep_apnea.png> |
| Set3_Msg8 | Video-based | Know the effects of using CPAP on drowsy driving. Watch this video:  <https://www.youtube.com/embed/d2UqGInReZ4> |
| Set3_Msg9 | Video-based | Sleep apnea could be hurting your heart. Use the CPAP to sleep well and be well!  <https://www.youtube.com/embed/vSxEvWjpqBo> |
| Set3_Msg10 | Image-based | Learn about how sleep disorders affect your heart:  <https://www.adventisthealth.org/images/import/wp-content/uploads/2016/03/sleep-disorders-infographic.png> |
| Set3_Msg11 | Video-based | Want to know what causes sleep apnea? Find out in this short video:  <https://www.youtube.com/embed/2JJExD2n39U> |
| Set3_Msg12 | Text-based | Do you have problems concentrating and staying awake during the day? Regular use of CPAP will help you get a better nights sleep so you can be awake during the day. |

***Set IV final messages***

| **Message** | **Mode** | **Content** |
| --- | --- | --- |
| Set4_Msg1 | Image-based | Learn how sleep apnea affects your whole body!  <https://www.whywesnore.com/x/lc-content/uploads/2017/02/2016-sleep-apnea-infographic-8-2.png> |
| Set4_Msg2 | Text-based | Sleep apnea can increase the risk of motor vehicle and workplace accidents. Using a CPAP device for at least 4 hours a night can help you to reduce this risk! |
| Set4_Msg3 | Image-based | Learn about the risks of untreated sleep apnea. Use your CPAP! Your health is your wealth!  <https://www.verywellhealth.com/thmb/eyDOJ3PGeHv5x161dCOTZ34VpbE=/1500x1000/filters:no_upscale():max_bytes(150000):strip_icc()/sleep-apnea-overview-3014774-v1-5c5dbe9a46e0fb00017dd118.png> |
| Set4_Msg4 | Text-based | Untreated obstructive sleep apnea increases your risk of high blood pressure. Use your CPAP for better health! |
| Set4_Msg5 | Image-based | Learn about the effects of untreated sleep apnea:  <https://static1.squarespace.com/static/591f6ccfb8a79bb177f17924/t/598b34ccd2b8578f996350f7/1506117076239/sleep-apnea-infographic-sleep-data.jpg?format=1500w> |
| Set4_Msg6 | Video-based | Watch this video to learn more about sleep apnea:  <https://www.youtube.com/embed/lu-pMXDZ8IA> |
| Set4_Msg7 | Text-based | Untreated obstructive sleep apnea increases your risk of heart problems. Use your CPAP for better health! |
| Set4_Msg8 | Image-based | Don’t wait. Act now:  https://atlantaheadachetmjpain.com/wp-content/uploads/untreated-obstructive-sleep-apnea-has-negative-effects-on-medical-conditions..png |
| Set4_Msg9 | Video-based | Want to learn more about sleep apnea and the benefits of using CPAP? Watch this video:  <https://www.youtube.com/embed/8_6CDuSiajA> |
| Set4_Msg10 | Text-based | Do you feel sleepy or tired during the day? Poor sleep may be a reason for sleepiness or tiredness. Use CPAP for at least 4 hours a night to feel better! |
| Set4_Msg11 | Video-based | Obstructive sleep apnea can make you feel tired all the time. Find out more in this video:  <https://www.youtube.com/embed/6bqTOfwvWhk> |
| Set4_Msg12 | Video-based | Sleep apnea could be hurting your heart. Use the CPAP to sleep well and be well!  https://www.youtube.com/embed/vSxEvWjpqBo |
